# Supplementary material for: 39Ar dating with small samples provides new key constraints on ocean ventilation
Source: Nat Commun. 2018 Nov 28;9:5046. doi: 10.1038/s41467-018-07465-7 (PMC6261945; doi:10.1038/s41467-018-07465-7)
Supplement: Supplementary file 1 — Supplementary Information [file 41467_2018_7465_MOESM1_ESM.pdf]

^{39}Ar dating with small samples provides new key constraints on ocean ventilation

Ebser et al.

**profile**	**longitude** [°W]	**latitude** [°N]	**pressure** [dbar]	**salinity PSS-78**	**temperature** [°C]	**weight** [kg]	**extracted Ar amount** [mL(STP)]	**number of measurements**	**total time** [h]	**counted atoms**	**^{39}Ar** [pmAr]
44	-22.99	11.55	4013	34.889	2.38	22.1	7.5	2	39.7	147	$47.1^{+5.4}_{-5.0}$
44	-22.99	11.55	3001	34.923	2.74	22.2	6.6	3	59.8	162	$35.0^{+4.4}_{-4.1}$
44	-22.99	11.55	1498	34.954	4.34	24.1	6.1	2	30.0	103	$46.2^{+6.2}_{-5.7}$
44	-22.99	11.55	997	34.807	5.62	22.0	6.8	3	60.1	231	$54.7^{+5.3}_{-4.9}$
44	-22.99	11.55	447	35.061	9.93	22.5	5.3	2	42.3	216	$78.7^{+8.0}_{-7.4}$
44	-22.99	11.55	398	35.100	10.41	23.1	5.7	2	40.0	217	$80.3^{+7.8}_{-7.2}$
44	-22.99	11.55	348	35.191	11.21	22.0	6.5	2	40.1	212	$74.9^{+7.3}_{-6.8}$
44	-22.99	11.55	12	35.909	24.95	20.7	5.2	2	34.9	241	$95.2^{+9.7}_{-9.0}$
55	-22.00	11.25	4006	34.888	2.38	22.5	7.2	2	40.8	104	$36.0^{+5.0}_{-4.5}$
55	-22.00	11.25	3000	34.922	2.73	24.0	lost during preparation				
55	-22.00	11.25	1500	34.962	4.30	24.0	6.4	2	40.0	147	$55.3^{+6.4}_{-5.9}$
55	-22.00	11.25	849	34.758	5.99	22.0	6.4	2	43.1	181	$61.5^{+6.2}_{-5.7}$
55	-22.00	11.25	448	35.096	9.79	22.0	6.0	2	47.2	215	$66.4^{+6.8}_{-6.2}$
55	-22.00	11.25	398	35.155	10.47	24.0	5.9	2	40.0	210	$78.9^{+7.9}_{-7.3}$
55	-22.00	11.25	348	35.190	10.99	22.0	5.5	2	40.0	220	$80.7^{+7.9}_{-7.3}$
55	-22.00	11.25	9	35.877	26.52	21.2	5.0	2	40.0	265	$98.5^{+9.1}_{-8.5}$
82	-24.28	17.58	3498	34.907	2.51	25.0	5.4	2	40.0	108	$36.0^{+5.4}_{-5.0}$
82	-24.28	17.58	2998	34.929	2.77	10.5	2.7	might be contaminated			
82	-24.28	17.58	2998	34.929	2.77	10.0	3.1	might be contaminated			
82	-24.28	17.58	1998	34.978	3.66	25.5	5.2	2	39.8	131	$46.4^{+6.1}_{-5.6}$
82	-24.28	17.58	1498	35.012	4.66	25.0	7.8	2	40.0	153	$54.1^{+6.3}_{-5.8}$
82	-24.28	17.58	796	34.918	7.07	9.0	lost during transport				
82	-24.28	17.58	796	34.918	7.07	10.0	2.3	1	15.7	71	$51.1^{+8.8}_{-7.9}$
82	-24.28	17.58	298	35.437	12.15	26.3	7.3	2	40.3	210	$78.2^{+8.7}_{-8.0}$

Supplementary Table 1 ^{39}Ar-results of the depth profiles 44, 55 and 82 taken during the cruise M116. The given uncertainties correspond to 1σ.

**profile**	**pressure** [dbar]	**^{39}Ar** [pmAr]	**CFC-12** [fmol kg^{-1}]	Δ/Γ	**mean age Γ** [a]	$C_{\mathbf{ant,\ best}}$ [µmol kg^{-1}]	$C_{\mathbf{ant,\ best}} - C_{\mathbf{ant},\Delta/\Gamma=1}$ [µmol kg^{-1}]
44/55	4009	$41.9^{+3.8}_{-3.6}$	13 ± 6	$0.8^{+0.3}_{-0.2}$	530^{+251}_{-131}	$3.4^{+0.8}_{-0.8}$	0.3
44	3001	$35.0^{+4.4}_{-4.1}$	8 ± 6	$1.0^{+0.7}_{-0.4}$	810^{+1193}_{-318}	$2.5^{+0.9}_{-0.7}$	0.0
44/55	1499	$51.2^{+4.6}_{-4.3}$	18 ± 6	$0.6^{+0.2}_{-0.1}$	331^{+109}_{-69}	$4.9^{+1.4}_{-1.1}$	1.2
44/55	923	$57.7^{+4.3}_{-4.0}$	16 ± 6	$0.5^{+0.1}_{-0.1}$	245^{+59}_{-43}	$6.2^{+1.6}_{-1.4}$	2.4
44/55	447	$72.1^{+5.5}_{-5.2}$	432 ± 18	$1.0^{+0.5}_{-0.3}$	166^{+121}_{-56}	$19.5^{+4.9}_{-4.0}$	0.0
44/55	398	$79.6^{+5.9}_{-5.5}$	548 ± 21	$0.7^{+0.4}_{-0.3}$	100^{+65}_{-37}	$24.2^{+7.3}_{-5.4}$	1.3
44/55	348	$77.7^{+5.7}_{-5.3}$	687 ± 25	$1.1_{-0.5}$	131_{-53}	$26.7^{+6.3}_{-5.1}$	−0.6
44/55	10	$97.0^{+7.1}_{-6.6}$	1018 ± 34				
82	3498	$36.0^{+5.4}_{-5.0}$	8 ± 6	$0.9^{+0.8}_{-0.3}$	744^{+1308}_{-297}	$2.6^{+1.1}_{-0.8}$	0.1
82	1998	$46.4^{+6.1}_{-5.6}$	6 ± 6	$0.6^{+0.2}_{-0.3}$	384^{+202}_{-113}	$3.6^{+1.5}_{-1.2}$	1.1
82	1498	$54.1^{+6.3}_{-5.8}$	16 ± 6	$0.6^{+0.2}_{-0.1}$	286^{+116}_{-71}	$5.3^{+2.3}_{-1.7}$	1.7
82	796	$51.1^{+8.8}_{-7.9}$	99 ± 9	$1.1_{-0.4}$	483_{-223}	$7.9^{+3.8}_{-2.9}$	−0.4
82	298	$78.2^{+8.7}_{-8.0}$	815 ± 28	$1.6_{-1.0}$	156_{-98}	$31.2^{+10.4}_{-8.2}$	−1.1

Supplementary Table 2 Data for the TTD-calculations: The combined data are the average of profile 44 and 55. The CFC-12 values are extrapolated along constant density from neighbouring profiles. The uncertainties of C_{ant} are calculated assuming the best estimate for Δ/Γ and taking the uncertainty range for ^{39}Ar into account. All given uncertainties correspond to 1σ.
